# Integrating genome-wide association study with regulatory SNP annotation information identified candidate genes and pathways for schizophrenia

**DOI:** 10.18632/aging.102008

**Published:** 2019-06-07

**Authors:** Xiao Liang, Sen Wang, Li Liu, Yanan Du, Bolun Cheng, Yan Wen, Yan Zhao, Miao Ding, Shiqiang Cheng, Mei Ma, Lu Zhang, Xin Qi, Ping Li, Xiong Guo, Feng Zhang

**Affiliations:** 1 Key Laboratory of Trace Elements and Endemic Diseases of National Health and Family Planning Commission, School of Public Health, Health Science Center, Xi’an Jiaotong University, Xi’an, China

**Keywords:** schizophrenia, regulatory SNP, genome-wide association studies, gene ontology (GO), pathways

## Abstract

Background: Schizophrenia is a complex mental disorder. The genetic mechanism of schizophrenia remains elusive now.

Methods: We conducted a large-scale integrative analysis of two genome-wide association studies of schizophrenia with functional annotation datasets of regulatory single-nucleotide polymorphism (rSNP). The significant SNPs identified by the two genome-wide association studies were first annotated to obtain schizophrenia associated rSNPs and their target genes and proteins, respectively. We then compared the integrative analysis results to identify the common rSNPs and their target regulatory genes and proteins, shared by the two genome-wide association studies of schizophrenia. Finally, DAVID tool was used to conduct gene ontology and pathway enrichment analysis of the identified targets genes and proteins.

Results: We detected 53 schizophrenia-associated target genes for rSNP, such as FOS (*P value* = 2.18×10^-20^), ATXN1 (*P value* = 5.22×10^-21^) and HLA-DQA1 (*P value* = 1.98×10^-10^). Pathway enrichment analysis identified 24 pathways for transcription factors binding regions, chromatin interacting regions, long non-coding RNAs, topologically associated domains, circular RNAs and post-translational modifications, such as hsa05034:Alcoholism (*P value* = 2.57×10^-7^) and hsa04612:Antigen processing and presentation (*P value* = 6.82×10^-8^).

Conclusion: We detected multiple candidate genes, gene ontology terms and pathways for schizophrenia, supporting the functional importance of rSNPs, and providing novel clues for understanding the genetic architecture of schizophrenia.

## INTRODUCTION

Schizophrenia (SCZ) is a chronic and severe mental disorder, characterized by psychosis, apathy and withdrawal, and cognitive impairment. Depression, anxiety, and substance abuse are additional mental health problems of SCZ condition. The symptoms of SCZ typically appear gradually, start between ages 16 and 30, and never resolve in many cases. About 1% of the population is affected by SCZ during their lifetimes, which is associated with substantial morbidity and mortality, as well as personal and societal costs [1, 2]. Moreover, SCZ is ranked among the top 25 leading causes of disability worldwide [3].

SCZ is a multi-factorial disease, and its occurrence depends on environmental and genetic factors. The high heritability of SCZ points to a major role for inherited genetic variants in the etiology of SCZ, with estimated heritability up to 80% [1, 2]. In recent years, considerable progress has been done in the genetic studies of SCZ [4]. Multiple susceptibility genes have been identified for SCZ [2, 5], such as NRG1 [5], DISC1 [5] and DRD2 [2]. Major histocompatibility complex (MHC) [1] on chromosome 6p is the most significant region for SCZ, which contains many markers reaching genome-wide significance [4]. However, the genetic mechanism of SCZ remains largely unknown.

Genome-wide association studies (GWAS) have great power to identify susceptibility genetic loci associated with complex diseases. Over the years, a number of creditable candidate genes for SCZ has been identified, largely by GWAS [2]. Because a stringent threshold requires a huge sample size to reliably identify risk genes, the significant loci identified by GWAS are usually limited and functionally independent, providing limited information for the mechanism studies of SCZ. Moreover, a large part of genetic variants detected by GWAS are located in non-coding chromosomal regions [4]. It is usually confusing how these non-coding regions implicated would be involved in the development of SCZ.

Functional SNPs located in protein-coding and non-coding genes are named regulatory single nucleotide polymorphisms (rSNPs), which usually have major impacts on gene functions [6]. They mainly include transcription factor binding regions (TFBRs), chromatin interactive regions (CIRs), topologically associated domains (TADs), long non-coding RNAs (lncRNAs) coding regions, and circular RNA (circRNA) regions. Additionally, some SNPs located in the protein coding regions can alter protein post-translational modifications (PTMs) [7], such as phosphorylation, methylation, acetylation, ubiquitination, and glycosylation. The implication of rSNPs in the development of complex diseases has been well documented in previous studies [8–10]. Integrating GWAS and functional rSNPs annotation information have improved GWAS power and provided novel clues for the genetic studies of complex diseases [11–13], such as periodontal diseases [13] and breast cancer [12]. To the best of our knowledge, limited efforts have been paid to explore the functional relevance of rSNPs with SCZ.

In this study, we conducted a large-scale integrative analysis of two GWAS datasets of SCZ with functional annotation datasets of rSNPs. The significant SNPs identified by the two GWAS were first annotated to obtain SCZ-associated rSNPs and their target gene and proteins, respectively. We then compared the integrative analysis results to identify the common rSNPs and their target gene and proteins, shared by the two GWAS of SCZ. Finally, DAVID tool was used to conduct gene ontology (GO) and pathway enrichment analysis of the identified target genes and proteins shared by the two GWAS of SCZ.

## RESULTS

### Analysis results of GWAS and rSNP annotation datasets

For TFBRs, CIRs, lncRNAs regions, TADs and circRNAs, we identified 1,499 SCZ associated rSNPs, corresponding to 35 genes, such as FOS (*P value* = 2.18×10^-20^), GABBR1 (*P value* = 2.18×10^-20^), MDK (*P value* = 1.89×10^-10^) and ATXN1 (*P value* = 5.22×10^-21^). For PTM, we detected 43 rSNPs, corresponding to 18 genes, such as HLA-DQA1 (*P value* = 1.98×10^-10^), HLA-DRB1 (*P value* = 1.36×10^-12^) and ZSCAN31 (*P value* = 8.78×10^-10^) (Table 1).

**Table 1:** List of SCZ associated rSNPs and their target regulatory genes and proteins shared by both SCZ 1 and SCZ 2.

**SNP**	**Gene**	**SNP-related regulatory elements**	***P value***
rs35001169	HIST1H3J	TFBRs, CIRs, lncRNAs regions, TADs, circRNAs	7.40× 10^-27^
rs35001169	HIST1H2AM	TFBRs, CIRs, lncRNAs regions, TADs, circRNAs	7.40× 10^-27^
rs35819751	MIR3143	TFBRs, CIRs, lncRNAs regions, TADs, circRNAs	9.52× 10^-27^
rs66462181	HIST1H4A	TFBRs, CIRs, lncRNAs regions, TADs, circRNAs	1.06× 10^-26^
rs17695758	DNAH8	TFBRs, CIRs, lncRNAs regions, TADs, circRNAs	1.52× 10^-26^
rs141342723	HIST1H2BL	TFBRs, CIRs, lncRNAs regions, TADs, circRNAs	1.82× 10^-26^
rs13209332	HIST1H2AK	TFBRs, CIRs, lncRNAs regions, TADs, circRNAs	2.10× 10^-26^
rs2232423	ZSCAN12	PTMs	1.71× 10^-23^
rs33932084	PGBD1	PTMs	5.00× 10^-23^
rs41266839	BTN3A1	PTMs	4.77× 10^-22^
rs34788973	OR2B2	PTMs	1.87× 10^-21^
rs34197618	ATXN1	TFBRs, CIRs, lncRNAs regions, TADs, circRNAs	5.22× 10^-21^
rs41266779	FOS	TFBRs, CIRs, lncRNAs regions, TADs, circRNAs	2.18× 10^-20^
rs41266779	HIST1H2BK	TFBRs, CIRs, lncRNAs regions, TADs, circRNAs	2.18× 10^-20^
rs41266779	NUP153	TFBRs, CIRs, lncRNAs regions, TADs, circRNAs	2.18× 10^-20^
rs41266779	PKHD1	TFBRs, CIRs, lncRNAs regions, TADs, circRNAs	2.18× 10^-20^
rs41266779	CLIC5	TFBRs, CIRs, lncRNAs regions, TADs, circRNAs	2.18× 10^-20^
rs41266779	DCDC2	TFBRs, CIRs, lncRNAs regions, TADs, circRNAs	2.18× 10^-20^
rs41266779	GABBR1	TFBRs, CIRs, lncRNAs regions, TADs, circRNAs	2.18× 10^-20^
rs41266779	PRIM2	TFBRs, CIRs, lncRNAs regions, TADs, circRNAs	2.18× 10^-20^
rs41266779	ANKH	TFBRs, CIRs, lncRNAs regions, TADs, circRNAs	2.18× 10^-20^
rs41266779	HIST1H2AH	TFBRs, CIRs, lncRNAs regions, TADs, circRNAs	2.18× 10^-20^
rs35050608	MBOAT1	TFBRs, CIRs, lncRNAs regions, TADs, circRNAs	2.37× 10^-20^
rs35506517	LY86	TFBRs, CIRs, lncRNAs regions, TADs, circRNAs	3.25× 10^-20^
rs9393718	HIST1H2BJ	TFBRs, CIRs, lncRNAs regions, TADs, circRNAs	3.32× 10^-20^
rs35555795	BTN1A1	PTMs	9.83× 10^-17^
rs79780963	RNU1-60P	TFBRs, CIRs, lncRNAs regions, TADs, circRNAs	7.51× 10^-16^
rs13216828	BTN3A2	PTMs	1.59× 10^-15^
rs9986596	ZKSCAN4	PTMs	5.51× 10^-15^
rs3891176	HLA-DQB1	PTMs	3.29× 10^-14^
rs11693528	BMPR2	TFBRs, CIRs, lncRNAs regions, TADs, circRNAs	5.62× 10^-14^
rs16891235	HIST1H1A	PTMs	1.59× 10^-13^
rs769949	PLCL1	TFBRs, CIRs, lncRNAs regions, TADs, circRNAs	2.10× 10^-13^
rs853678	ZSCAN31	PTMs	3.42× 10^-13^
rs281786	MPP4	TFBRs, CIRs, lncRNAs regions, TADs, circRNAs	3.92× 10^-13^
rs281786	AOX1	TFBRs, CIRs, lncRNAs regions, TADs, circRNAs	3.92× 10^-13^
rs35220450	RAPH1	TFBRs, CIRs, lncRNAs regions, TADs, circRNAs	4.05× 10^-13^
rs35220450	INO80D	TFBRs, CIRs, lncRNAs regions, TADs, circRNAs	4.05× 10^-13^
rs281760	AOX2P	TFBRs, CIRs, lncRNAs regions, TADs, circRNAs	4.71× 10^-13^
rs10734901	ATP6V0A2	TFBRs, CIRs, lncRNAs regions, TADs, circRNAs	4.83× 10^-13^
rs3098341	BOLL	TFBRs, CIRs, lncRNAs regions, TADs, circRNAs	4.89× 10^-13^
rs10431750	KLC1	TFBRs, CIRs, lncRNAs regions, TADs, circRNAs	5.69× 10^-13^
rs56155997	PDE11A	TFBRs, CIRs, lncRNAs regions, TADs, circRNAs	5.92× 10^-13^
rs34525648	SLC17A2	PTMs	1.22× 10^-12^
rs16822516	HLA-DRB1	PTMs	1.36× 10^-12^
rs71417869	CDC42BPB	TFBRs, CIRs, lncRNAs regions, TADs, circRNAs	4.02× 10^-12^
rs2075800	HSPA1L	PTMs	6.00× 10^-11^
rs35324223	MDK	TFBRs, CIRs, lncRNAs regions, TADs, circRNAs	1.89× 10^-10^
rs707962	HLA-DQA1	PTMs	1.98× 10^-10^
rs115817940	HLA-DRB5	PTMs	3.46× 10^-10^
rs13201753	ZKSCAN3	PTMs	8.78× 10^-10^
rs950169	ADAMTSL3	PTMs	9.17× 10^-10^
rs13107325	SLC39A8	PTMs	5.03× 10^-9^

Note: TFBRs, transcription factor binding regions; CIRs, chromatin interactive regions; TADs, topologically associated domains; PTMs, protein post-translational modifications.

### GO enrichment analysis

GO enrichment analysis identified 15 GO terms enriched in the identified target genes of TFBRs, CIRs, lncRNAs, TADs and circRNAs, such as GO:0000786~nucleosome (*P value* = 1.84×10^-10^), GO:0046982~protein heterodimerization activity (*P value* = 5.97×10^-7^), GO:0000788~nuclear nucleosome (*P value* = 5.63×10^-5^) and GO:0006334~nucleosome assembly (*P value* = 5.70×10^-5^). For PTMs, we identified 37 SCZ-associated GO terms, such as GO:0002504~antigen processing and presentation of peptide or polysaccharide antigen via MHC class II (*P value* = 4.79×10^-7^), GO:0042613~MHC class II protein complex (*P value* = 1.03×10^-6^), GO:0042605~peptide antigen binding (*P value* = 2.26×10^-6^) and GO:0071556~integral component of lumenal side of endoplasmic reticulum membrane (*P value* = 2.43×10^-6^) (Table 2).

**Table 2:** List of SCZ associated gene ontology terms shared by both SCZ 1 and SCZ 2.

**Term**	**Term description**	**SNP-related regulatory elements**	***P value***
GO:0000786	nucleosome	TFBRs, CIRs, lncRNAs regions, TADs, circRNAs	1.84× 10^-10^
GO:0002504	Antigen processing and presentation of peptide or polysaccharide antigen via MHC class II	PTMs	4.79× 10^-7^
GO:0046982	protein heterodimerization activity	TFBRs, CIRs, lncRNAs regions, TADs, circRNAs	5.97× 10^-7^
GO:0042613	MHC class II protein complex	PTMs	1.03× 10^-6^
GO:0042605	Peptide antigen binding	PTMs	2.26× 10^-6^
GO:0071556	Integral component of lumenal side of endoplasmic reticulum membrane	PTMs	2.43× 10^-6^
GO:0030658	Transport vesicle membrane	PTMs	5.57× 10^-6^
GO:0030669	Clathrin coated endocytic vesicle membrane	PTMs	7.03× 10^-6^
GO:0002381	Immunoglobulin production involved in immunoglobulin mediated immune response	PTMs	8.50× 10^-6^
GO:0050852	T cell receptor signaling pathway	PTMs	9.71× 10^-6^
GO:0012507	ER to Golgi transport vesicle membrane	PTMs	1.45× 10^-5^
GO:0002455	Humoral immune response mediated by circulating immunoglobulin	PTMs	1.78× 10^-5^
GO:0019882	Antigen processing and presentation	PTMs	1.81× 10^-5^
GO:0030666	Endocytic vesicle membrane	PTMs	2.98× 10^-5^
GO:0060333	Interferon gamma mediated signaling pathway	PTMs	3.90× 10^-5^
GO:0031295	T cell costimulation	PTMs	5.17× 10^-5^
GO:0000788	nuclear nucleosome	TFBRs, CIRs, lncRNAs regions, TADs, circRNAs	5.63× 10^-5^
GO:0006334	nucleosome assembly	TFBRs, CIRs, lncRNAs regions, TADs, circRNAs	5.70× 10^-5^
GO:0032588	Trans Golgi network membrane	PTMs	5.92× 10^-5^
GO:0019886	Antigen processing and presentation of exogenous peptide antigen via MHC class II	PTMs	8.46× 10^-5^
GO:0032395	MHC class II receptor activity	PTMs	8.78× 10^-5^
GO:0003677	DNA binding	TFBRs, CIRs, lncRNAs regions, TADs, circRNAs	2.20× 10^-4^
GO:0005737	cytoplasm	TFBRs, CIRs, lncRNAs regions, TADs, circRNAs	5.83× 10^-4^
GO:0043565	Sequence specific DNA binding	PTMs	1.19× 10^-3^
GO:0005765	Lysosomal membrane	PTMs	1.96× 10^-3^
GO:2001179	Regulation of interleukin 10 secretion	PTMs	2.86× 10^-3^
GO:0006342	chromatin silencing	TFBRs, CIRs, lncRNAs regions, TADs, circRNAs	2.91× 10^-3^
GO:0032673	Regulation of interleukin 4 production	PTMs	3.81× 10^-3^
GO:0072643	Interferon gamma secretion	PTMs	6.65× 10^-3^
GO:0006955	Immune response	PTMs	6.87× 10^-3^
GO:0003700	Transcription factor activity, sequence specific DNA binding	PTMs	1.10× 10^-2^
GO:0042088	T helper 1 type immune response	PTMs	1.14× 10^-2^
GO:0016020	Membrane	PTMs	1.17× 10^-2^
GO:0016045	Detection of bacterium	PTMs	1.23× 10^-2^
GO:0002437	Inflammatory response to antigenic stimulus	PTMs	1.42× 10^-2^
GO:0000139	Golgi membrane	PTMs	1.64× 10^-2^
GO:0031047	gene silencing by RNA	TFBRs, CIRs, lncRNAs regions, TADs, circRNAs	1.67× 10^-2^
GO:0042405	nuclear inclusion body	TFBRs, CIRs, lncRNAs regions, TADs, circRNAs	2.19× 10^-2^
GO:0032689	Negative regulation of interferon gamma production	PTMs	2.64× 10^-2^
GO:0035774	Positive regulation of insulin secretion involved in cellular response to glucose stimulus	PTMs	2.73× 10^-2^
GO:0016021	Integral component of membrane	PTMs	2.81× 10^-2^
GO:0010507	Negative regulation of autophagy	PTMs	3.38× 10^-2^
GO:0007040	Lysosome organization	PTMs	3.38× 10^-2^
GO:0042130	Negative regulation of T cell proliferation	PTMs	3.47× 10^-2^
GO:0051262	Protein tetramerization	PTMs	3.75× 10^-2^
GO:0005654	nucleoplasm	TFBRs, CIRs, lncRNAs regions, TADs, circRNAs	3.77× 10^-2^
GO:0005887	Integral component of plasma membrane	PTMs	3.81× 10^-2^
GO:0007214	gamma-aminobutyric acid signaling pathway	TFBRs, CIRs, lncRNAs regions, TADs, circRNAs	3.86× 10^-2^
GO:0070062	extracellular exosome	TFBRs, CIRs, lncRNAs regions, TADs, circRNAs	3.98× 10^-2^
GO:0000790	nuclear chromatin	TFBRs, CIRs, lncRNAs regions, TADs, circRNAs	4.24× 10^-2^
GO:0002227	innate immune response in mucosa	TFBRs, CIRs, lncRNAs regions, TADs, circRNAs	4.38× 10^-2^
GO:0032200	telomere organization	TFBRs, CIRs, lncRNAs regions, TADs, circRNAs	4.72× 10^-2^

Note: TFBRs, transcription factor binding regions; CIRs, chromatin interactive regions; TADs, topologically associated domains; PTMs, protein post-translational modifications.

### Pathway enrichment analysis

For TFBRs, CIRs, lncRNAs, TADs and circRNAs, we identified 3 pathways associated with SCZ, including ha05322:Systemic lupus erythematosus (*P value* = 3.77×10^-8^), hsa05034:Alcoholism (*P value* = 2.57×10^-7^) and hsa05203:Viral carcinogenesis (*P value* = 1.78×10^-2^). For PTMs, we identified 21 pathways associated with SCZ, such as hsa04612:Antigen processing and presentation (*P value* = 6.82×10^-8^), hsa05310:Asthma (*P value* = 7.44×10^-7^), hsa05332:Graft-versus-host disease (*P value* = 1.00×10^-6^) and hsa04672:Intestinal immune network for IgA production (*P value* = 2.96×10^-6^) (Table 3).

**Table 3:** List of SCZ associated pathways shared by both SCZ 1 and SCZ 2.

**Term description**	**Term ID**	**SNP-related regulatory elements**	***P value***
Systemic lupus erythematosus	hsa05322	TFBRs, CIRs, lncRNAs regions, TADs, circRNAs, PTMs	3.37× 10^-8^
Antigen processing and presentation	hsa04612	PTMs	6.82× 10^-8^
Alcoholism	hsa05034	TFBRs, CIRs, lncRNAs regions, TADs, circRNAs	2.57× 10^-7^
Toxoplasmosis	hsa05145	PTMs	3.06× 10^-7^
Asthma	hsa05310	PTMs	7.44× 10^-7^
Graft-versus-host disease	hsa05332	PTMs	1.00× 10^-6^
Allograft rejection	hsa05330	PTMs	1.42× 10^-6^
Influenza A	hsa05164	PTMs	1.94× 10^-6^
Type I diabetes mellitus	hsa04940	PTMs	2.10× 10^-6^
Intestinal immune network for IgA production	hsa04672	PTMs	2.96× 10^-6^
Autoimmune thyroid disease	hsa05320	PTMs	4.03× 10^-6^
Staphylococcus aureus infection	hsa05150	PTMs	4.52× 10^-6^
Viral myocarditis	hsa05416	PTMs	5.33× 10^-6^
Inflammatory bowel disease (IBD)	hsa05321	PTMs	7.58× 10^-6^
Leishmaniasis	hsa05140	PTMs	1.04× 10^-5^
Rheumatoid arthritis	hsa05323	PTMs	1.99× 10^-5^
Epstein-Barr virus infection	hsa05169	PTMs	5.30× 10^-5^
Systemic lupus erythematosus	hsa05322	PTMs	7.03× 10^-5^
Cell adhesion molecules (CAMs)	hsa04514	PTMs	8.36× 10^-5^
Phagosome	hsa04145	PTMs	9.84× 10^-5^
Tuberculosis	hsa05152	PTMs	1.61× 10^-4^
Herpes simplex infection	hsa05168	PTMs	1.78× 10^-4^
HTLV-I infection	hsa05166	PTMs	4.71× 10^-4^
Viral carcinogenesis	hsa05203	TFBRs, CIRs, lncRNAs regions, TADs, circRNAs	1.78× 10^-2^

Note: TFBRs, transcription factor binding regions; CIRs, chromatin interactive regions; TADs, topologically associated domains; PTMs, protein post-translational modifications.

## DISCUSSION

Considering that most of SCZ variants identified by GWAS were not causal, integration of the GWAS results with functional rSNPs information is a powerful approach to discover novel candidate genes for SCZ [4]. To evaluate the roles of rSNPs in the pathogenesis of SCZ, we conducted a large-scale integrative genomics analysis of two GWAS datasets of SCZ with functional annotation datasets of rSNPs. We identified multiple candidate genes, GO terms, and biological pathways for SCZ. Our study results support the functional importance of rSNPs in the genetic mechanism of SCZ, and provide novel clues for understanding the genetic architecture of SCZ.

We identified several candidate genes for SCZ, such as FOS, GABBR1, MDK, ATXN1 and ZSCAN31. FOS is classified as one of the immediate early genes (IEGs), which encode not only transcription factors, but also a much wider variety of proteins, including signaling molecules, growth factors and cytoskeletal proteins [14]. Alteration in the expression of IEGs is linked to neuron generation and neuronal cell death. Nadia Cattane et al. have reported that FOS was significantly upregulated in the fibroblasts of SCZ patients [14]. Defects in synaptic plasticity are involved in the pathophysiology of SCZ. Interestingly, SNPs either protective (rs1063169) or positively associated with SCZ (rs7101T) were identified, showing transcription factor c-fos was important in the regulation of synaptic plasticity [15]. Huang et al. observed high FOS expression in non-neural peripheral samples and low FOS expression in the brain tissues of SCZ patients compared with healthy controls, which suggests that FOS is a sensitive marker for SCZ [16]. In addition, detection of FOS in blood samples may be helpful for SCZ diagnosis [16]. These combined results support the functional relevance of FOS with SCZ [14–16], which is consistent with our study results.

GABBR1 is another SCZ-associated gene identified by this study. γ-aminobutyric acid_B_ (GABA_B_) is an inhibitory transmitter molecule acting at neuronal synapses. Functional GABA_B_ receptor requires both the GABBR1 and GABBR2 subunits. GABA_B_ receptor can modulate the release of a number of neurotransmitters, including dopamine, serotonin, noradrenaline, somatostatin, glutamate and GABA [17]. Interestingly, the reduction of GABBR1 in pyramidal cells, and consequent reduction of GABA_B_ receptors, can result in the dysfunction of inhibitory mechanism and increase signal output [18]. Previous studies have also observed association between GABBR1 and SCZ. Fatemi et al. observed a significant reduction of GABBR1 protein in the lateral cerebellum of the subjects with SCZ, bipolar disorder, and major depression [17]. In addition, Zai et al. conducted a case-control study and detected a positive association between GABBR1 and SCZ [19].

Genetic variation in a region on chromosome 11 that contains MDK was significantly associated with SCZ [20]. In addition, MDK also accumulated in senile plaques in the hippocampus of patients with Alzheimer’s disease [20, 21]. Notably, ATXN1 serves as one of the hub genes in the protein-protein interaction network containing many known SCZ risk genes [22]. Actually, ATXN1 is highly expressed in prefrontal cortex, and SCZ patients had significantly decreased ATXN1 expression [22]. In addition, Takeo Saito et al. have revealed an association of rs7779855 in ZSCAN31 with SCZ [23].

GO analysis detected several GO terms for SCZ. One important finding of this study is the disclosure of the MHC class II protein complex (GO:0042613), a class of MHC molecules like human leukocyte antigens HLA-DQA1 and HLA-DQB1, which present antigens to CD4-positive T-lymphocytes. The association between SCZ and the immune system has been repeatedly revealed in genetic, epidemiological and post mortem studies [24]. The protein encoded by HLA-DQA1 gene binds to the protein encoded by HLA-DQB1. Together, they form a functional protein complex called an antigen-binding DQαβ heterodimer, which displays foreign peptides to immune system to trigger body's immune response. Interestingly, HLA alleles were previously shown to be associated with SCZ risk [25]. rs9272105 within HLA-DQA1 explained 1–3% of variation in attentional control, and to a lesser extent, premorbid intelligence quotient in psychotic patients [26]. Additionally, rs9272105 within HLA-DQA1 was also individually associated with variation in neuropsychological function [26]. It has been demonstrated that the level of HLA-DRB1 in SCZ was decreased in peripheral blood samples in contrast with increased level in prefrontal cortex [27]. Moreover, HLA-DPA1 and CD74, which are integral components of the MHC Class II protein complex, were both reduced in hippocampus, amygdala, and dorsolateral prefrontal cortex regions in SCZ [28].

T helper 1 type immune response (GO:0042088) is another significant GO term detected by this study. Delayed hypersensitivity reaction is the classic cell-mediated immune response with sensitized T helper-1 cells. Michael et al. found an attenuated skin reactivity to various antigens in SCZ patients [29]. They also observed significantly diminished responses of schizophrenics to antigen stimulation with tetanus and diptheria antigen presentation [29]. Significantly increased serum level of interleukin-18, a cytokine known to activate T helper 1 type cells in immune responses, has been previously observed in SCZ patients [30].

In addition, we also identified several other GO terms associated with SCZ, such as Golgi membrane (GO:0000139) and GABA signaling pathway (GO:0007214). Previously, it was shown that differentially expressed genes related to Golgi apparatus, vesicular transport and membrane association were over-represented in SCZ [31]. In line, Devor et al. identified a large number of GABA-associated genes for SCZ [32].

The involvement of cell adhesion molecules (CAMs) in the pathophysiology of SCZ has long been hypothesized. In this study, CAMs (hsa04514) pathway was detected for SCZ. The CAMs pathway is implicated in a variety of neurocognitive processes, including memory and attention-related reaction time. Multiple CAM genes has been reported to be associated with SCZ risk [26]. Neural CAMs are very important members of the exclusive group of the molecules responsible for precise wiring of nervous system. Neural CAMs serve as ‘‘glue’’ in cell-to-cell adhesion and contact-mediated attraction [33]. They can interact with numerous matrix components, and are involved in many aspects of neuronal development, synaptogenesis, and myelination, which guide brain morphology and support highly-coordinated brain activity [33]. Cerebrospinal fluid neuronal CAMs were significantly increased in SCZ patients [34]. Additionally, the plasma levels of intercellular adhesion molecule-1 was also elevated in SCZ patients [35]. Honer et al. found that syntaxin immunoreactivity in the cingulate cortex from schizophrenics was increased, along with neural CAMs and the CAMs to synaptophysin ratio [36]. Besides, it has been reported that L1 cell adhesion molecule interaction was involved in neuronal function [37].

Another interesting SCZ associated pathway is alcoholism (hsa05034). Recent studies have suggested that alcoholism has a wide-reaching influence on the clinical course of SCZ, contributing to shape abnormalities in hippocampus and subcortical shape differences [38]. We also observed that systemic lupus erythematosus (SLE) (hsa05322) was associated with SCZ. Despite the fact that SCZ is not classified as a typical autoimmune diseases, the dysregulation of the immune system cannot be excluded [39, 40]. Interestingly, DNA and myelin basic protein (MBP)-hydrolyzing antibodies, which play an important harmful role in SLE pathogenesis, were also detected in the sera of SCZ patients. In addition, light chains of IgGs from SCZ patients were similar to those of SLE patients [41].

The majority of SNPs identified by GWAS are enriched in non-coding regions [4], and contribute to complex traits and diseases through various molecular mechanisms. These include effects on transcription factor binding affinities, which can result in differential gene expression [11]. However, significant loci identified by GWAS have rarely been tracked to causal polymorphisms thus far. Integrative analysis of GWAS with functional rSNPs is helpful to improve GWAS power and provide novel clues for pathogenetic studies of SCZ. Notably, the functional SNPs data can assist to exclude unlikely genes/loci, effectively reducing the number of tests needed for unbiased searches across the genome, thus, improving the power to discover novel causal loci [4]. To the best of our knowledge, this is the first large-scale integrative analysis of GWAS and rSNPs for SCZ. The implication of rSNPs in the development of SCZ was systematically investigated considering TFBRs, CIRs, lncRNAs regions, TADs, circRNAs, and PTMs in our study. Nevertheless, one limitation of this study should be noted. The SCZ-associated SNP sets were driven from previous GWAS. The accuracy of our integrative analysis may be affected by the power of previous GWAS of SCZ. Therefore, further studies are warranted to confirm our findings.

In conclusion, we conducted a large-scale integrative genomics analysis of two GWAS datasets of SCZ with functional annotation datasets of rSNPs to explore the genetic basis of rSNPs in the pathogenesis of SCZ. We observed multiple candidate genes, GO terms and pathways for SCZ. We hope that our study results could provide novel clues for the pathogenic and therapic studies of SCZ.

## MATERIALS AND METHODS

### The first GWAS dataset of SCZ (SCZ1)

A large GWAS meta-analysis data of SCZ was driven from the Psychiatric Genomics Consortium (PGC) [42], totally containing 33,426 SCZ cases and 32,541 controls. Genotypes from all studies were processed by the PGC using unified quality control procedures followed by imputation of SNPs and insertion-deletions using the 1000 Genomes Project reference panel [43]. Quality control and imputation were performed on each of the study cohort datasets, according to the standard procedures established by the PGC [42]. Genotype imputation was performed using the pre-phasing/imputation stepwise approach implemented in IMPUTE2 [44] and SHAPEIT [45]. The imputation reference set consists of 2,186 phased haplotypes from the full 1000 Genomes Project dataset. Logistic regression was conducted to control for 13 components of ancestry, study sites and genotyping platform. Detailed description of sample characteristics, experimental design, statistical analysis and quality control can be found in the previous studies [42].

### The second GWAS dataset of SCZ (SCZ2)

Another independent GWAS data of SCZ [2] was used here. Briefly, this GWAS included 36,989 SCZ cases and 113,075 controls, from 49 ancestry matched, non-overlapping case-control samples (46 of European and three of East Asian ancestry, 34,241 cases and 45,604 controls) and 3 family-based samples of European ancestry (1,235 parent affected-offspring trios). Genotypes from all studies were processed by the PGC using unified quality control procedures. The 1000 Genomes Project reference panel was used for SNPs imputation [43]. In each sample, association testing was conducted using imputed SNP dosages and principal components to control for population stratification. After quality control (imputation INFO score ≥ 0.6, MAF ≥ 0.01, and successfully imputed in ≥ 20 samples), they considered around 9.5 million variants. An inverse-weighted fixed effects model was used for final meta-analysis. Detailed description of sample characteristics, experimental design, statistical analysis and quality control can be found in the previous study [2].

### rSNPs annotation datasets

The rSNPs annotation information were driven from the rSNPBase 3.1 database (http://rsnp3.psych.ac.cn) [46] and the AWESOME database (http://www.awesome-hust.com) [7]. rSNPBase 3.1 provided rich functional annotation for human SNP-related regulatory elements and their target regulatory genes, including TFBRs, CIRs, mature microRNA (miRNA) regions, predicted miRNA target sites, lncRNA regions, TADs and circRNAs. AWESOME database is an analysis tool that systematically evaluates the role of SNPs on nearly all kinds of PTMs based on 20 available tools. They construct a comprehensive platform to collect and integrate SNPs and multiple PTM information, utilizing 24 published database or tools. 1,043,608 germline missense variants from the dbSNP was used and each SNP was matched with its protein sequence in AWESOME. Detailed description of the two rSNPs annotation database can be found in the published studies [7, 46].

### Statistical analysis

The significant SNPs with GWAS *P value* < 5.0 × 10^-8^ were selected from the two GWAS of SCZ (SCZ1 and SCZ2). The selected SCZ-associated SNPs were then annotated by the rSNPBase 3.1 database [46] and the AWESOME database to obtain SCZ associated rSNPs and their target regulatory genes and proteins. We then compared the integrative analysis results to identify the common rSNPs and their target genes and proteins shared by the two GWAS of SCZ. To explore the functional relevance of identified target regulatory genes and proteins with SCZ, GO and pathway enrichment analyses of the identified common target genes and proteins shared by the SCZ1 and SCZ2 were performed by the Database for Annotation, Visualization and Integrated Discovery (DAVID) tool [47].
